# Dynamically tunable switch and filter in single slot cavity structure

**DOI:** 10.1038/s41598-019-51192-y

**Published:** 2019-10-10

**Authors:** Boxun Li, Lili Zeng, Xingjiao Zhang, Biao He, Kun Liao, Kun Liu, Bin Wang

**Affiliations:** 1 School of Mechatronics Engineering, Ping Xiang University, Ping Xiang, 337055 P.R. China; 2 College of Solar Energy Engineering, Hunan Vocational Institute of Technology, Xiangtan, 411104 P.R. China; 3 College of Physics and Electronics, Central South University, Changsha, 410083 P.R. China

**Keywords:** Optical sensors, Nanophotonics and plasmonics

## Abstract

A single slot cavity coupled with two waveguides has been researched in theory and simulation. The results comparison between theory and simulation shows they agree well. It is found that the lateral displacement *S* plays an important role in transmission properties. Moreover, increasing the width of the slot cavity results in the emergence of new resonant peaks. At the same time, the shift of the resonant peaks have been explained well. The slot cavity with Kerr nonlinear material can act as a dynamically tunable four channel switch and filter. The single slot cavity has the advantages of simple and compact structure, easy fabrication, and the excellent properties are helpful to control light in photonics circuits.

## Introduction

Surface plasmons (SPs) are electromagnetic waves coupled to free electron oscillations traveling along the metal-dielectric interface. Due to the capabilities of overcoming diffraction limit and confining light in deep sub-wavelength dimension^1^. SPs provides possibility for nanodevices with high miniaturization and integration^2^. Consequently, the SPs is considered as a promising way for realization of highly integrated optical circuits^3–8^.

The metal-dielectric-metal (MDM) waveguide structure, with the remarkable advantages of long propagation distance, easy fabrication and convient integration, has attracted tremendous attentions^9,10^. Some researchers had investigated the tunable plasmonic structures based on MDM waveguide. It can also be applied in sensor and filter^11–17^. For example, Xu *et al*. studied the effect of nano defect in stub on transmission properties^13^. Zhan *et al*. researched Fano resonance in MDM waveguide which can be acted as a tunable nanoplasmonic sensor^14^. Li *et al*. reported Fano resonance based on multimode and degenerate mode interference^16^. Huang *et al*. reported the sensing performance of the MDM waveguide which is based on transparency effect can be enhanced^17^. For dynamically tunable with different structures, the traditional realization method is to directly adjust the parameters. However, to a given structure, once the plasmonic nanostructure has been fabricated, it is unrealistic to achieve adjustable the properties by changing the parameters. More efforts still need to be made to realize the dynamic adjustable devices.

In this paper, a single slot cavity has been investigated theoretically and numerically. The simulated results agree well with the the theoretical results. The lateral displacement *S* plays an important role in the transmission property. The first and second order mode can be separated without interference by choosing an appropriate *S*. Attributing to the superposition of resonant modes, it is found that new resonant peaks emerge by increasing the width of slot cavity. The shift of the resonant peaks have been explained well. The results reveal that the single slot cavity with Kerr nonlinear material can act as a dynamically tunable four channel switch and filter. This work paves a new way towards the realization of dynamic control of light in photonics circuits.

## Model and Method

**Figure 1:**
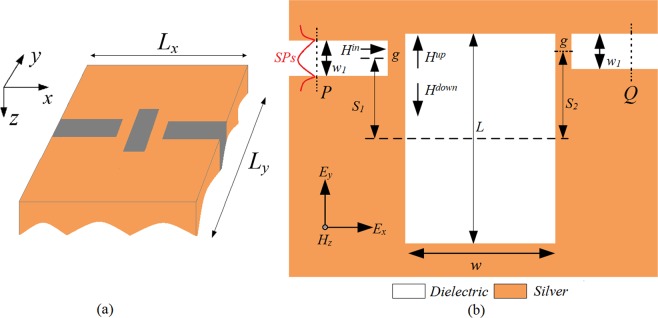
The (**a**)schematic and (**b**)top view of the 2D single slot cavity waveguide coupling structure.

The proposed single slot cavity waveguide coupling structure is schematically shown in Fig. 1, which consists of a slot cavity coupled with two waveguides, and the single slot cavity waveguide coupling structure is a 2-D metallic structure which is infinitely long in z-axis. The incident light is along the x direction with TM polarization (the magnetic field is parallel to z direction). TM-polarized wave is emitted from *P* and propagates to *Q*. *P*_P_ and *P*_*Q*_ stand for input and output power flows, respectively. The transmission is defined as *T = P*_*Q*_*/P*_P_. The width of waveguides and slot cavity are set to be *w*_1_ and *w*, where the width of waveguides *w*_1_ equal to 50 nm. *L* stands for the length of slot cavity. *S*_1_ and *S*_2_ present the lateral displacement between the central of two waveguides and reference plane of slot cavity. The distance between slot cavity and two waveguides is *g*, which is a constant. Here, the orange and white areas are silver and dielectric, respectively. Unless otherwise specified, the dielectric is air in this manuscript. The Drude model *ε*_*m*_(*ω*) = *ε*_*∞*_ − *ω*_*p*_*/*(*ω*^2^ + *iωγp*) is introduced to characterize the complex relative permittivity of silver, where the dielectric constant *ε*_*∞*_ = 3.7 at the infinite frequency, the bulk plasma frequency *ω*_*p*_ = 1.38 × 10^16^rad/s, *ω* stands for the angle frequency of incident wave, the damping rate *γ*_*p*_ = 2.73 × 10^13^rad/s characters the absorption loss^18^. The finite-difference time-domain (FDTD) with perfectly matched layer (PML) absorbing boundary is introduced to investigate the configuration. The computational space is *L*_*x*_ × *L*_*y*_ = 1000 *nm* × 1000 *nm*, the spatial and temporal steps are set as Δ*x* = Δ*y* = 5 *nm*, and Δ*t* = Δ*x*/2*c* (c is the velocity of light in vacuum), respectively, which are sufficient for the convergence of numerical results.

## Results and Discussions

When SPs are coupled into the slot cavity, we define *φ* to be the phase delay per round-trip in slot cavity, and *φ* has the relation^19^ *φ* = 4π*n*_*eff*_*L*/*λ*_*m*_ + *φ*_*r*_, where *n*_*eff*_, *λ*_*m*_ and *φ*_*r*_ are the effective refractive index, resonance wavelength and phase shift of a beam reflected on the upper and lower facets of slot cavity, respectively. The *φ*_*r*_ is very little which has no obvious effect on the resonant wavelength. And the effective refractive index can be obtained from the dispersion equation^20–22^:

1 $${{\varepsilon }}_{{m}}\sqrt{{{{n}}_{{eff}}}^{{2}}-{{\varepsilon }}_{{d}}}\,{\tanh }(\frac{w{\pi }\sqrt{{{{n}}_{{eff}}}^{{2}}-{{\varepsilon }}_{{d}}}}{{\lambda }}){+}{{\varepsilon }}_{{d}}\sqrt{{{{n}}_{{eff}}}^{{2}}{-}{{\varepsilon }}_{{m}}}=0\,$$

**Figure 2:**
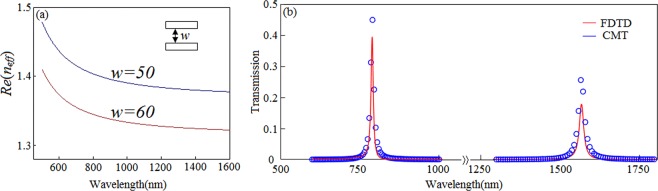
(**a**) Effective refractive index as function of wavelength with various *w* (nm). (**b**) Simulated (red line) and theoretical results (blue cycle) with *S*_1_ = *S*_2_ = 275 nm, *L* = 600 nm and *w* = 50 nm.

Where *ε*_*m*_ and *ε*_*d*_ stand for the dielectric constant of metal and dielectric waveguide with a width of *w*, respectively. Figure 2(a) shows *n*_*eff*_ as a function of wavelength with various *w*. It is found that the value has significant various in the visible range. With the increasing of wavelength, the curve becomes relatively flatter when the wavelength increases from 800 nm to 1600 nm. Moreover, it is demonstrated from Fig. 2(a) that the wider the width and the smaller the value of *n*_*eff*_ at a given wavelength.

For the single cavity waveguide coupling structure, a temporal coupled mode theory (CMT) is introduced to research the transmission features. In order to simplify the calculation, we neglect the propagation and coupling losses as well as the phase shift, the temporal normalized mode amplitude *a* of slot cavity can be expressed as^23^

2 $$\frac{da}{dt}=(-j{\omega }_{0}-{k}_{0}-{k}_{1}-{k}_{2})a+{A}_{+1}\sqrt{{k}_{1}}+{A}_{+2}\sqrt{{k}_{2}}$$

Where *ω*_0_ represents the resonance frequency of the slot cavity, *k*_0_, *k*_1_ and *k*_2_ respectively stand for the internal loss of slot cavity and the coupling loss to two waveguides, which satisfy the relationships *k*_*i*_ = *ω*_0_/2*Q*_*i*_ (*i* = 0, 1, 2). Here *Q*_*i*_ are the related quality factors. *Q*_1_ and *Q*_2_ are controlled by the coupling distances g, which has been proved in some reference^24^. And there is a contradiction between quality factor and transmittance. That is the transmittance decreases and the quality factor increases with the increasing of distance *g*. According to the energy conservation, the transmission *T* of the single cavity waveguide coupling structure can be derived as^25^

3 $${T}={|\frac{\sqrt{2{k}_{1}{k}_{2}}}{{j}({\omega }-{{\omega }}_{0}){+}{k}_{0}{+}{k}_{1}{+}{k}_{2}}|}^{{2}}$$

Figure 2(b) describes the simulated and theoretical results which are presented by red line and blue cycle, respectively. Most obviously, the simulated results agree well with theoretical results. A little deviation can be attributed to the assumptions of neglecting coupling and propagation losses as well as phase shift.

**Figure 3:**
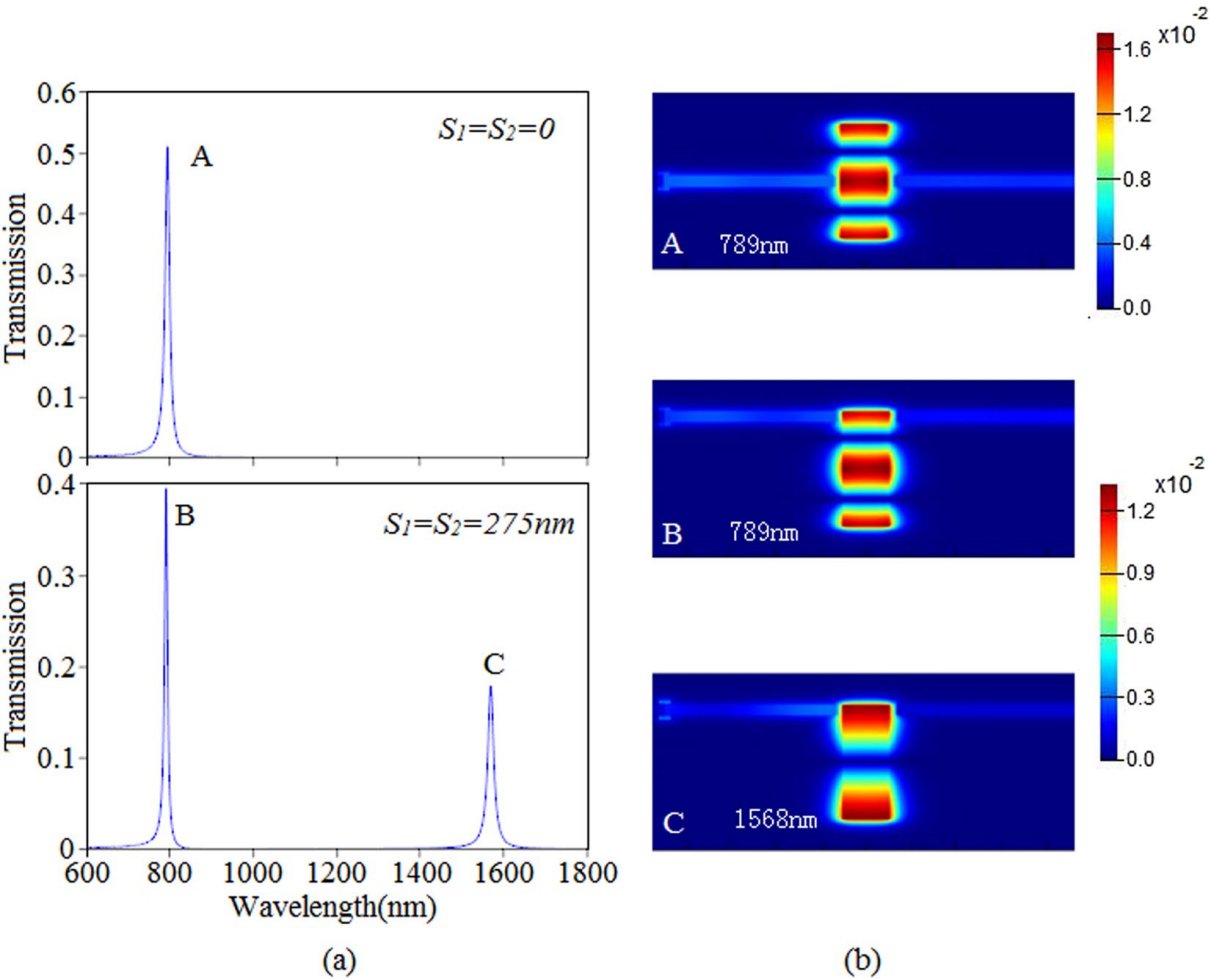
(**a**) The transmission spectral with different *S*_1_ and *S*_2_. (**b**) The magnetic fields of three peaks A, B and C.

Figure 3(a) shows the transmission spectral with various distance between the centre of two waveguides and reference plane of slot cavity. When *S*_1_ = *S*_2_ = 0, it is found that there is a transmission peak A which locates at 789 nm. However, when *S*_1_ = *S*_2_ = 275 nm, there are two transmission peaks B and C which are located at 789 nm and 1568 nm, respectively. What needs to be pointed out is that the transmittance of peak A is about 52% which is higher than that of peak B with a transmittance 39%. In order to investigate the abnormal phenomenon, we have plot the magnetic fields of peak A, B and C, which are illustrated in Fig. 3(b). Obviously, the distributions of fields of A and B are similar. And the intensity of A is stronger than that of B. It is concluded from magnetic fields of peak A that the stable standing waves has been excited in the slot cavity when the resonant condition is satisfied, and the peak A is the second order mode of the slot cavity. Similarly, the magnetic fields of B and C correspond to the second and the first order mode, respectively. According to magnetic fields, due to coupled resonance between slot cavity and waveguide, it is concluded that the energy can indirectly couple into output waveguide when the output waveguide locates at the high energy areas of the slot cavity. It is obvious that the first order mode do not exist in the slot cavity when *S*_1_ = *S*_2_ = 0, while the first order and the second order mode can exist in the slot cavity when *S*_1_ = *S*_2_ = 275 nm. Based on the results above, we can derive that the second order mode can couple into output waveguide while the first order mode cannot when *S*_1_ = 275 nm and *S*_2_ = 0.

Based on the superposition principle of optics^26^, we qualitatively analyze the transmission spectral with different *S*_1_ and *S*_*2*_. When the resonance condition is satisfied^19^: *Φ* = *β*_*m*_*·2 L* + *θ = 2mπ*, where *θ* stands for the phase shift of a beam reflected on the up and down facets of the slot cavity. *β*_*m*_ is the propagation constant of SPs corresponding to the *m*^st^ order resonance mode of the slot cavity. Just as shown in Fig. 1, the input field *H*^*in*^ inside the slot cavity is divided into two parts, the *H*^*up*^ and *H*^*down*^, which have the relation of *H*^*up*^ = *H*^*down*^* = H*^*in*^*/*2 = *H*_0_ and propagate in opposite directions. Intensity inside the slot cavity with an arbitrary input position *S* can be expressed as:

4 $${H}_{m}(x,t)=\frac{2{H}_{0}\,\cos ({\beta }_{m}x-0.5{\beta }_{m}L)}{\sigma }\times \{\exp [j(1.5{\beta }_{m}L-{\beta }_{m}{S}_{1})]+\exp [j({\beta }_{m}{S}_{1}+0.5{\beta }_{m}L)]\}\,\times \exp (-j{\omega }_{m}t)$$

Where σ stands for the dissipation of the light propagating. Neglect the phase shift *θ*, one has *Φ* = *β*_*m*_*·2* *L* = *2mπ*, for the first order mode (*m* = 1), the Eq. (3) can be expressed as

5 $${H}_{1}(x,t)=2{H}_{0}\,\cos ({\beta }_{1}x-0.5\pi )\times [-\,2\,\sin ({\beta }_{1}{S}_{1})]\times \exp (-\,j{\omega }_{1}t)/\sigma $$

For the second order mode (*m* = 2), the Eq. (3) can be written as:

6 $${H}_{2}(x,t)=\,2{H}_{0}\,\cos ({\beta }_{2}x-\pi )\times [-\,2\,\cos ({\beta }_{2}S)]\times \exp (-\,j{\omega }_{2}t)/\sigma $$

According to the equations above, it is concluded that when *S*_1_ = 0, *H*_1_ = 0, *H*_2_ ≠ 0, that is to say, the first order mode cannot exist in the slot cavity, while the second order mode can exist when *S*_1_ = 0. At the same time, when *S*_1_ = 275 nm, the first and the second order mode can exist in the slot cavity. The equation helps explain why different displacement results in different transmission property. What is worth mentioning is that the transmission spectral of the single cavity waveguide coupling structure has a high quality factor (QF), where the QF of peak B and C are 94 and 98, respectively. These excellent characters can be applied in filter.

**Figure 4:**
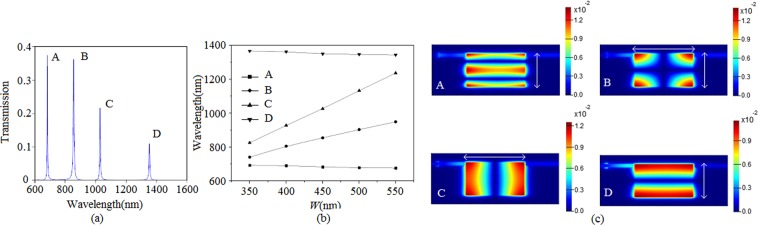
(**a**) The transmission spectrum with *w* = 450 nm, where other parameters are *S*_1_ = *S*_2_ = 275 nm and *L* = 600 nm. (**b**) The wavelengths of A, B, C and D with different width *w*. (**c**) The magnetic fields of A, B, C and D in (**a**).

In order to investigate the effect of parameters on transmission spectrum, we change the width of the slot cavity, it is found that four peaks appear in the spectrum when the width increases to 350 nm. Obviously, a phenomenon with two new peaks emerges in the Fig. 4(a). As is shown in Fig. 4(b), it is seen that the peaks B and C have a obvious red shift with the increasing of width *w*, while the peaks A and D have a opposite variation tendency. What is noteworthy is that the variation tendencies of peaks B and C are greater than that of A and D. To explore the underlying mechanism, just as illustrated in Fig. 4(c), the magnetic fields of A, B, C and D in Fig. 4(a) are plot. Obviously, the fields of peaks A and D correspond to the second and the first order mode, respectively, which are similar to the Fig. 3(b). And the energy propagate along the vertical direction of the slot cavity which is shown by the white double arrow in Fig. 4(c). To the peak C, it is found that the energy propagate along the horizontal direction of the slot cavity. It is concluded from magnetic field of B that the SPs propagate along the vertical and horizontal directions in the slot cavity, which can be deemed to the superposition of C and D. To the peaks A and D, due to the SPs propagate along vertical direction, the propagation length is *L*, and the width is *w*. According to the the phase delay equation (*φ* = 4π*n*_*eff*_*L*/*λ*_*m*_ + *φ*_*r*_) and dispersion Eq. (1), increasing the width *w* means decreasing the *n*_*eff*_, which results in the decrease of resonant wavelength, and the transmission spectrum manifests a blue shift. Moreover, according to the Fig. 2(a), increasing width *w* has not great impact on the *n*_*eff*_, and the variation tendencies of peaks A and D change little. To the peak C, the propagation length is *w* and the width is *L*, increasing *w* will increase the propagation length, and the resonant wavelength *λ*_*m*_ will shift to long wavelength. With respect to the peak B, the propagation length is *w* + *L*^27^, the resonant wavelength also shift to long wavelength. In reference^27^, there is a similar conclusion to ours. To the four peaks A, B, C and D, they can be calculated quantificationally, due to the high QF, the properties can be also applied in filter.

In order to realize the function of dynamic regulation to the single slot cavity, the two waveguides and slot cavity are filled with a Kerr nonlinear material whose nonlinear refractive index can be adjustable with an external control (pumping) beam. Due to large third-order optical nonlinear susceptibility χ^(3)^ and ultra-fast response time which are important for switching function, Au/SiO_2_ with χ^(3)^ = 1.7 × 10^−7^esu (2.37 × 10^−15^m^2^/v^2^)^28^ is chosen to fill in the slot cavity and two waveguides. The refractive index of is expressed as *n* = *n*_0_ + *n*_2_*I*^29^, where *n*_0_ = 1.47 is the linear refractive index, *n*_2_ = 2.07 × 10^−9^cm^2^/W is the nonlinear refractive index coefficient and *I* is the pumping beam intensity.

**Figure 5:**
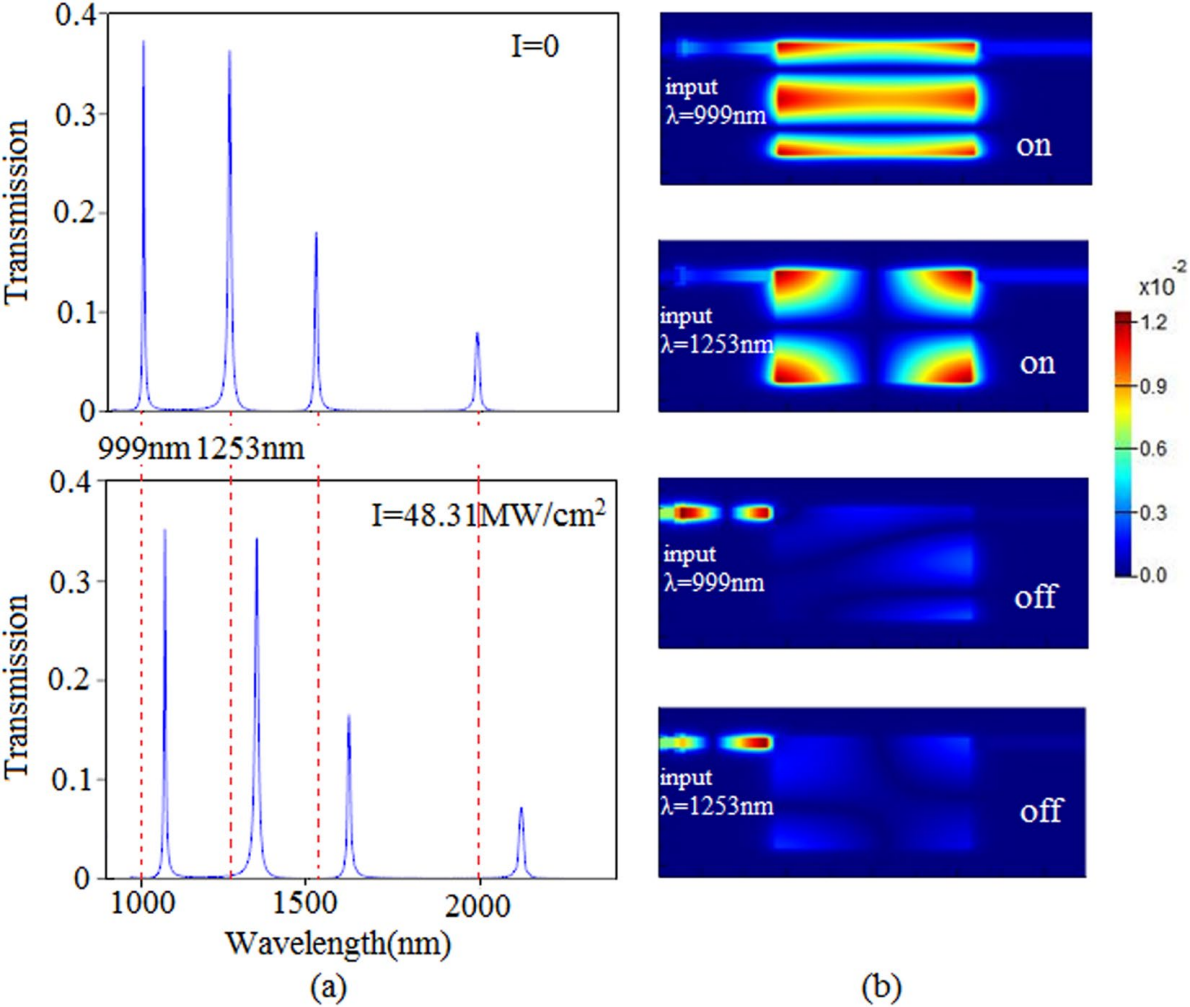
(**a**) The transmission spectra at two different pumping intensities with *L* = 600 nm, *w* = 450 nm. (**b**) The magnetic fields at wavelengths of 999 nm and 1253 nm. The ‘on’ state without the pumping light and the ‘off’ state with the pumping light. The dielectric is Kerr nonlinear material.

Figure 5(a) shows the transmission spectra at two different pumping intensities. It is found that the four peaks shift to long wavelength with changing the pumping intensity *I*. When the pumping intensity *I* equals to 0, the wavelength 999 nm with a transmittance of 37% can pass through the output port. With the pumping intensity *I* increasing to 48.31 MW/cm^2^, the transmittance of wavelength 999 nm tends to 0 and cannot couple into output port. Likewise, the other three wavelengths have the similar variation tendency. Figure 5(b) displays the magnetic fields of 999 nm and 1253 nm without and with pumping light. It can be seen that the light can couple into output port without pumping light at the wavelengths of 999 nm and 1253 nm. These results can be regarded as the ‘on’ state. Moreover, when pumping light with *I* = 48.31 MW/cm^2^ is introduced, the light cannot couple into the output port, which can be regarded as the ‘off’ state. Table 1 describes the different wavelengths stay ‘on/off’ state with and without pumping light. To a given wavelength, it is clear that the light can or cannot couple into output port when *I* = 0 or *I* = 48.31 MW/cm^2^. In other words, the single slot cavity can be applied in dynamically tunable multi-switching by introducing pumping light. These excellent properties have an important application in optical circuits.

**Table 1:** The different wavelengths stay on/off state with and without pumping light.

λ(nm)	I = 0		I = 48.31 MW/cm^2^	
	Transmittance	State	Transmittance	State
999	0.37	on	0.009	off
1066	0.004	off	0.35	on
1253	0.36	on	0.007	off
1340	0.007	off	0.35	on
1517	0.18	on	0.008	off
1610	0.003	off	0.17	on
1989	0.12	on	0.005	off
2115	0.006	off	0.1	on

## Conclusion

In sum, a single slot cavity has been investigated theoretically and numerically. The simulated results agree well with the theoretical results. The lateral displacement *S* plays an important role in the transmission property. The first and second order mode can be separated without interference by choosing an appropriate *S*. Due to the superposition of resonant modes, it is found that new resonant peaks emerge by increasing the width of slot cavity. The shift of the resonant peaks have been explained well. The results reveal that the single slot cavity with Kerr nonlinear material can act as a dynamically tunable four channel switch and filter. The proposed single slot cavity has the advantages of simple and compact structure, easy fabrication, which paves a new way towards the realization of dynamic control of light in photonics circuits.
